# Evaluation of psychiatric symptoms in NMDA receptor encephalitis in predicting clinical deterioration and mortality: a systematic review and qualitative synthesis

**DOI:** 10.1002/alz70857_103453

**Published:** 2025-12-25

**Authors:** Gina Eom, Julien Hébert, Nicholas Lum, Max Stern, Emerson Joseph Marinas, Corinne E. Fischer

**Affiliations:** ^1^ Hennick Bridgepoint Hospital, Sinai Health Systems, Toronto, ON, Canada; ^2^ University of Toronto, Toronto, ON, Canada; ^3^ University of Buckingham, Buckingham, England, United Kingdom; ^4^ Unity Health Toronto, Toronto, ON, Canada; ^5^ Keenan Research Centre for Biomedical Science, Li Ka Shing Knowledge Institute, St. Michael's Hospital, Toronto, ON, Canada; ^6^ Toronto Dementia Research Alliance, University of Toronto, Toronto, ON, Canada

## Abstract

**Background:**

NMDA receptor encephalitis is a rare limbic encephalitis where autoantibodies target autogenous receptors in the brain, resulting in symptoms including anxiety, restlessness, psychosis, dyskinesias, central hypoventilation and seizures. A significant proportion of affected individuals have associated germ‐cell tumours, or with viral infections presumably unmasking some sort of inflammatory process. Treatment often involves immunosuppression, and due to central hypoventilation can result in ICU stay. Here, we sought to examine whether these psychological and behavioural symptoms have prognostic clinical value.

**Method:**

Given the rare presentation of NMDA encephalitis, we first conducted a systematic review to find case reports, case series and cohort studies that contained individual patient level data which had both a descriptive data of the clinical presenting symptoms, with particular attention to psychiatric symptoms. We then performed both a quantitative and qualitative analysis of the studies that were included in the analysis. Patient level data was then analyzed quantitatively and qualitatively.

**Result:**

We analyzed over 500 cases published on NMDA receptor encephalitis, that had enough clinical descriptors of their presentation and outcomes. In particular, we observed trends with regards to demographics, clinical symptoms and clinical outcome. Preliminary data points towards an interesting association with psychiatric symptoms (such as paranoia) and clinical outcomes.

**Conclusion:**

Psychiatric syndromes may yield important prognostic value in NMDA encephalitis.

